# HPV Reflex Testing in Menopausal Women

**DOI:** 10.4061/2011/181870

**Published:** 2011-04-07

**Authors:** Emily M. Ko, Rosemary Tambouret, David Wilbur, Annekathryn Goodman

**Affiliations:** ^1^MGH/Vincent Department of Obstetrics and Gynecology, Division of Gynecologic Oncology, Massachusetts General Hospital, Boston, MA 02114, USA; ^2^Department of Pathology, Massachusetts General Hospital, Boston, MA 02114, USA

## Abstract

*Objective*. To determine the frequency of high risk (HR) HPV and intraepithelial neoplasia following ASCUS pap cytology screens in menopausal women. *Study Design*. Following IRB approval, we performed a retrospective review of all cases of ASCUS pap tests, HPV results, and relevant clinical-pathologic data in women age 50 or over from November 2005 to January 2007 within a tertiary care center. Statistical analyses were performed in EXCEL. *Results*. 344 patients were analyzed for a total of 367 screening pap tests. 25.29% (87/344) patients were HR HPV positive, with greater percentages of HR HPV cases occurring in women age 65–74. Within HR HPV cases, 79.3% (69/87) underwent colposcopy. 27.5% (19/69) biopsy proven lesions were discovered, including cervical, vulvar or vaginal (intraepithelial neoplasia). Within the negative HR HPV group 3.1% (8/257) patients were diagnosed with dysplasia or carcinoma. Within both HR HPV positive and negative groups, patients with no prior history of lower genital tract lesions or cancer were identified. *Conclusion*. Reflex HPV testing plays an important role in ASCUS triage in menopausal women. Pap test screening and HPV testing should not be limited to women of reproductive age as they may aid in the diagnosis of intraepithelial neoplasia in women of older age.

## 1. Introduction

Cervical cancer remains the fourth leading cause of cancer mortality in women worldwide today [1]. It is estimated that 12,200 new cases of cervical cancer will be diagnosed in the US in 2010, with 4210 deaths due to the disease [2]. Approximately 60% of cervical cancers occur in women over the age 45, and 20% in women over age 65 [3]. 

Most studies on cervical cancer screening have focused on premenopausal women, with little information provided on the perimenopausal or postmenopausal (PMP) population. However, these women remain at increased risk for cervical cancer given that there is a secondary peak in prevalence of high-risk HPV subtypes in this older population [4], and that certain high-risk HPV subtypes persist to a greater extent in this population [5]. Unfortunately, many of these older women have not had regular access to gynecologic care, or cervical cancer screening. Of menopausal women who were diagnosed with invasive cervical cancer, 50% of women over age 65 had not had a pap smear in the past three years [6]. Approximately, one quarter of elderly women have never been screened by pap testing [7]. 

Furthermore, there is very limited data available on HPV infection and lower genital tract disease in menopausal women. Given that high-risk (HR) HPV reflex testing has increased the detection of Cervical Intraepithelial Neoplasia (CIN) based on the US National Cancer Institute ASCUS-LSIL Triage Study [8], we seek to determine the rate of HPV-positive infection by reflex testing in menopausal women with abnormal pap cytology of Atypical Squamous Cells of Undetermined Significance (ASCUS). This study also seeks to determine the frequency of lower genital tract disease, including CIN, vulvar intraepithelial neoplasia (VIN), and vaginal intraepithelial neoplasia (VAIN) in this population.

## 2. Methods

This single-institution study was conducted after approval by and following guidelines set forth by the Institutional Review Board. Pap cytology tests were obtained by obstetrician-gynecologists, internal medicine primary care physicians, as well as gynecologic-oncologists. Tests were done primarily at a tertiary care center, with a small percentage of cases received from local affiliated community health centers.

Using a pathology database from November of 2005 to January 2007, all cases of ASCUS in women aged 50 or over were retrospectively identified. Starting in November 2005, all cases of ASCUS pap results automatically underwent reflex high-risk (HR) HPV testing using the *Digene Hybrid Capture II System* (Qiagen, Valencia, CA, 2003). The *Sure Path* (TriPath Imaging Inc, Burlington, North Carolina, 2003) system was used to process all cytology specimens. Follow-up colposcopy and biopsy results were obtained from the electronic medical record. These exams were performed by both general obstetrician-gynecologists, nurse practitioners supervised by obstetrician gynecologists, and gynecologic-oncologists. Additionally, demographic information including age, race, age of menopause, past medical history, and past gynecologic history was collected. 

Data was entered in Excel. Basic distribution statistics were performed in Excel.

## 3. Results

344 patients were analyzed, with a total of 367 ASCUS cases. There were 22 patients who were screened twice and one who was screened three times during this interval. 

The mean age of menopause was 49.5 (SD 22.5) years. The mean age of subjects was 59.8 (SD 8.7) years. 17.4% (60/344) of patients were past smokers, and 11.04% (38/344) currently smoke. Overall, 46.5% (160/344) are currently married. 12.2% (42/344) are currently divorced and single, and 10.5% (36/344) are divorced or widowed with a new sexual partner at the time of the exam. Hypertension was the most frequently reported medical condition, present in 33.1% (114/344) of all cases. Conditions that may affect HPV-related diseases, such as, lupus, hepatitis, and postorgan transplantation occurred infrequently. 

In review of past gynecologic history, 42.7% (147/344) of patients never had an abnormal pap smear. 44.8% (154/344) of patients had been previously diagnosed with ASCUS. 15.7% (54/344) had history of CIN, and 3.5% (12/344) had history of cervical carcinoma in situ (CIS) and squamous cell carcinoma (SCC). 8.4% (29/344) had history of VIN. 14.0% (48/344) had prior hysterectomies, for either malignant or benign indications. In total, 20.3% (70/344) had a documented history of CIN, VIN, or VAIN.

**Figure 1:**
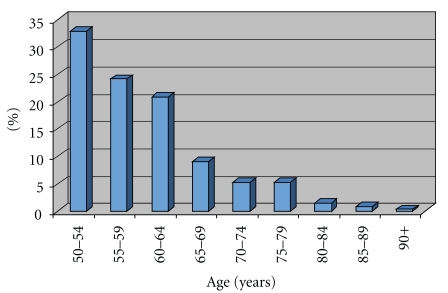
Age distribution of all ASCUS cases.

The majority of ASCUS cases occurred in women age 50–60, in which 56% of patients were less than 60 years old (Figure 1). With increasing age, there were fewer cases of ASCUS. 

**Figure 2:**
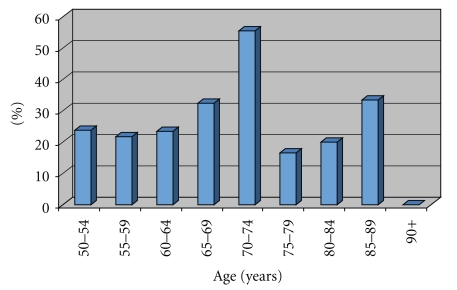
Percent of HPV+ ASCUS cases per age group.

Of these ASCUS samples, 25.3% (87/344) patients were HR HPV positive. This included all patients who on first pass were HR HPV positive, as well as one patient who was screened twice in this interval, who was first negative for HR HPV, and then positive. The greatest percent of HPV positive cases by age occurred within the age group of 65–74 group (Figure 2). 

Of patients who underwent repeated screens during our study period: 5 patients were negative on both screens (of which 1 patient was diagnosed with VAIN I). 12 patients were positive on both screens (of which one patient was diagnosed with CIN I, one with CIN2, and one with vulvar SCC). 1 patient was positive on 3 screens. 3 patients were positive on first screen, then converted to negative (of which 1 patient was diagnosed with VAIN III). 1 patient was negative initially, and then positive on repeat screen. 

Of the HPV positive patients, 79.3% (69/87) underwent colposcopy. 27.5% (19/69) of biopsy proven lesions were discovered, including CIN, VAIN, and VIN. Biopsy results revealed 7 patients with CIN I, 2 patients with CIN II, 2 patients with CIN III, 5 patients with VAIN and 1 patient with VIN.  Table 1 summarizes these findings. 

Of the HPV positive patients who did not initially receive colposcopy, further analysis showed that one patient underwent hysterectomy with no abnormal findings; 4 patients underwent colposcopy which were all negative; 3 patients underwent vaginal exam only (given prior hysterectomy) which were negative. 3 patients had no followup and are presumed to be lost to followup. The remaining patients all had at least repeat pap exams. 

Within the negative HR HPV group, 11.7% (30/257) patients underwent colposcopy. While current guidelines do not recommend colposcopy in HPV-negative women, in 2005, some women were still being treated based on older recommendations for colposcopy for atypical pap smears. Three percent of patients (8/257) were diagnosed with CIN, VIN, VAIN, or carcinoma. Findings included: one with CIN 1, one with VAIN II, one with VAIN III, one with newly diagnosed vaginal SCC, one with vulvar CIS, one with vulvar SCC, and 2 with newly diagnosed endometrial cancer.  Table 1 summarizes these findings with each individual's prior gynecologic history.

Overall, 7.8% of patients with ASCUS had positive colposcopic findings (19 HPV-positive plus 8 HPV-negative colposcopy biopsy proven cases/344). HPV triage allowed identification of 19/27 (70.3%) of the dysplastic or cancer cases.

## 4. Discussion

Despite the common assumption that peri- and postmenopausal (PMP) women are at low risk for lower genital tract disease, we found that 25% of ASCUS patients who were age 50 years or older had high-risk HPV. Within each five-year age bracket starting at 50, the greatest percentage of HR HPV occurred within women aged 60–70s. Furthermore, several of these women with biopsy proven diagnoses of lower genital tract disease had no prior history of HPV-related gynecologic disease. We presume that these cases are due to newly acquired infections. Without continued surveillance and gynecologic care, many PMP women may suffer from undiagnosed lower genital tract disease. 

Furthermore, 27% of HR HPV-positive patients were found to have a biopsy proven abnormality, including CIN, VIN, VAIN, or cancer. Even in the HPV-negative group, 3% of patients had biopsy proven disease, including two who have never had lower genital tract disease. They were newly diagnosed with endometrial cancer. Since we could not accurately differentiate women with a remote history of lower genital tract disease from those with more recent abnormalities, we did not exclude patients undergoing follow-up surveillance for recent abnormalities. Therefore, we reported all findings seen at this tertiary care center. Although these rates of lower genital tract abnormalities may be higher than that of the general PMP population, use of the pap cytology screening and HPV reflex testing helped identify a large proportion of PMP women with lower genital tract disease.

 Our results are consistent with other reports regarding the presence of lower genital tract disease, including HPV-related disease in PMP women. In a recent study of HR HPV A SCUS outcomes in PMP women age 45 years and older, 25.4% of ASCUS samples were HR HPV positive, similar to ours. Of these women, 30.9% revealed persistent LSIL/CIN1 and 3.68% were diagnosed with HSIL/CIN2or 3, with one patient diagnosed with squamous cell carcinoma (0.74%) [9]. Furthermore, in a recent study published from the Netherlands, the incidence of cervical cancer 10 years after having had three consecutive negative pap smears was similar for older women aged 45–55 (41/100,000, with 95% CI 33–51) versus younger women, aged 30–44 years (36/100,000 with 95% CI 24–52), *P* = .48 [10]. Contrary to common belief that the incidence of cervical cancer would decrease with increased age, the incidence of cancer remained nearly the same, in this large national cohort of women. Even after three negative pap tests, PMP women may warrant further surveillance.

Although guidelines for cervical cancer screening remain quite variable, our findings suggest that a significant proportion of PMP have new or persistent lower genital tract disease. Screening recommendations by various health organizations have been summarized in Table 2. Considerations for continued gynecologic examinations, pap cytology exam, and reflex HPV testing are important tools for identification and treatment of PMP women. In a rapidly growing elderly and PMP population, relatively little information is available regarding the frequency and occurrence of lower genital tract disease. The acquisition and persistence of HPV-related disease may differ in this population due to physiologic changes of menopause. Several PMP women had persistence of HR HPV while others cleared the infection on repeat exams in our study. Given the extent of lower genital tract disease found in our population compared to the relatively limited information reported about PMP women, we propose that the epidemiologic and social-behavioral patterns of PMP women may be underrecognized and underreported, both in regards to sexually transmitted infections and non-sexually transmitted diseases of the lower genital tract. 

The strengths of this study include that it was a single-institution study, with a designated gynecologic pathology department, diagnosing both cytologic as well as histologic specimens. The samples were aggregated from both community clinics affiliated with the institution as well as gynecologic-oncology subspecialty departments. The use of reflex testing was standardized during this time interval. 

Limitations of the study include the relatively small number of cases. Given that the occurrence of HPV-related genital disease is relatively uncommon, the frequencies reported in this study could be falsely elevated due to the referral base to a gynecologic oncology department, specialty colposcopy clinic, and gynecologic-pathology department within a large tertiary care center. Secondly, the actual incidence of HR HPV in this population is unknown. To our knowledge, the rate of lower genital tract disease in this older population remains undefined. 

In summary, PMP women remain susceptible to HPV related lower genital tract disease. Reflex HPV testing identified a significant fraction of patients with lower genital tract disease. Despite their increased age, these women should not be disregarded and should continue to undergo routine gynecologic exams, pap cytology testing, and reflex HPV testing in the appropriate clinical context. Reflex HPV remains a useful modality for identifying several treatable lower genital tract diseases in this older population.

## Figures and Tables

**Table 1:** Biopsy proven lesions following ASCUS cytology results.

	Biopsy proven	Prior gynecologic diagnoses
	Diagnosis	
HPV positive		

CIN I	7	4/7: No abnormal history
		2/7: LSIL
		1/7: CIN II

CIN II	2	1/2: LSIL
		1/2: CIN III

CIN III	2	1/2: No abnormal history
		1/2: AGUS

VAIN I	2	1/2: No abnormal history
		1/2: Cervical SCC

VAIN II	1	1/1: No abnormal history

VAIN III	2	1/2: Cold knife cone (pathology unavailable)
		1/2: CIN III

VIN III + SCC	1	1/1: Vulvar SCC

HPV negative		

CIN I	1	1/1: CIN 1
VAIN II	1	1/1: CIN I, VAIN I
VAIN III	1	1/1: CIN II, VAIN I
Vaginal SCC	1	1/1: No abnormal history
Vulvar CIS	1	1/1: Vulvar CIS
Vulvar SCC	1	1/1: Vulvar SCC
Endometrial cancer	2	2/2: No abnormal history

Biopsy proven results following ASCUS cytology, and reflex HR HPV testing. CIN. Cervical intraepithelial neoplasia. VAIN. Vaginal intraepithelial neoplasia. VIN. Vulvar intraepithelial neoplasia. CIS. Carcinoma in situ. SCC. Squamous cell carcinoma.

**Table 2:** Cervical cancer screening guidelines in PMP women.

Organization	Recommendation
American Congress of Obstetricians and Gynecologists	(i) Inconclusive evidence to set the upper age limit at which to stop cervical cancer screening
	(ii) Following hysterectomy for benign indications and with no prior history of CIN or cancer may discontinue screening

American Cancer Society	(i) Women ageing 70+ who had 3 consecutive negative pap screens and no abnormal screens within the past 10 years may stop screening, if they do not have a history of cancer, diesthylstilbesterol exposure, HIV, or suppressed immune status
	(ii) Following hysterectomy for benign indications and with no prior history of CIN or cancer may discontinue screening

United States Preventative Task Force	(i) Women of age 65+ with history of prior negative screens and who are deemed not at high risk for cervical cancer may stop cervical cancer screening
	(ii) Following hysterectomy for benign indications, cervical cancer screening can be stopped completely
